# Editorial

**DOI:** 10.1120/jacmp.v6i4.2214

**Published:** 2005-11-22

## Guest Editorial Celebrating the $90^{\text{th}}$ birthday of Dr. Moses Greenfield

We published a review of the electronic book “Review of Radiation Oncology Physics: A Handbook for Teachers and Students”, Edited by Ervin B. Podgorsak, PhD. in the JACMP Vol 5, Issue 3, 2004. A slightly revised version of this 695 page book is available without cost to anyone with web access. This new IAEA version is available on the web site: www.medphys.mcgill.ca/academic/IAEAsyllabus.pdf

It is with great pleasure I welcome as a Guest Editorial the following biography of Moses Greenfield by Dr. Amos Norman. This Fall 2005 issue of the JACMP is dedicated to Dr. Greenfield, and contains contributions from several of his former students.

Michael D. Mills, PhD

## MOSES A. GREENFIELD

Moses Greenfield was born in New York City on 8 March 1915. He earned a B.S. degree in physics from City College of New York where he also was awarded the Belden Medal in mathematics. In 1937 he received an M.S. degree in physics from New York University. and completed there all the requirements for the Ph.D. except the dissertation, He accepted an appointment as a Research Physicist during World War II at the Bureau of Ships, United States Navy, in Washington D.C. where, with others, he showed that a faulty design was responsible for the tendency of Liberty Ships to suddenly split in two. While working there he also was able, with the guidance of George Gamow, to write his Ph.D. dissertation on “The Problem of Energy Production in Red Giants” and was awarded the degree in 1941.

He left the Navy in 1946 for California and a position as a consultant for North American Aviation in Stellar Space Research. Then in 1948 he accepted an appointment as Associate Professor in the Department of Radiology in the new medical school under construction at the University of California, Los Angeles. He spent his entire career at UCLA since then, moving up in due course to Professor and Professor Emeritus. Early in his career of teaching, research and public service, in 1956, he founded with Amos Norman a graduate program in Medical Physics and served for many years as its director. This program, was later renamed the Interdepartmental Graduate Program in Biomedical Physics to reflect its large expansion to include the study and application of the many new devices – CT and Ultrasound Scanners, PET, SPECT and MRI – and procedures – IMRT, for example – made possible by the rapid evolution of computers.

This program, which to date has granted some 266 M.S. and Ph.D. degrees, is Professor Greenfield's major contribution to the Medical Physics Profession. Undoubtedly, this contribution, together with his many published papers in the field and, notably, the work with his graduate students and post docs on the measurement of bone density, influenced the American Association of Physicists in Medicine to give him its highest praise, the William D. Coolidge Award. There is every reason to expect that the next several decades will witness as many innovations in the practice of medical physics as in the past half century during which the slide rule was replaced by the computer. A reasonable guess is that it will witness more machine analysis of images and the incorporation of biological factors unique to each patient into radiation therapy treatment planning. The Biomedical Physics Graduate Program at UCLA is likely to flourish, and remain an enduring tribute to Professor Greenfield's leadership.

At 90 Moe Greenfield continues to attend and actively participate in meetings of the Board of the Emeriti Association and of the Faculty of the Biomedical Physics Program. He can be found frequently dining with his wife Bella, a mathematician, and friends at the UCLA Faculty Center where the conversation often turns to discussion of his children, stepchildren and 10 grandchildren (so far). I look forward to celebrating his one hundredth birthday in 2015 with his family, friends and colleagues.

Amos Norman

**Table:** 

The Journal of Applied Clinical Medical Physics	
**Editorial Board 2005**	
Editor‐in‐Chief	Michael D. Mills
Deputy Editor‐in‐Chief	Timothy D. Solberg
Associate Editor‐at‐Large	Richard Stark
**Associate Editors**	
Radiation Oncology Physics	Nzhde Agazaryan, B. Gino Fallone
	John Gibbons, Michael Herman
	Janelle Molloy, Matthew Podgorsak
	Nikos Papanikolaou
	Mehrdad Sarfaraz, Lu Wang
Medical Imaging	William Pavlicek
Radiation Measurements	Larry DeWerd
Radiation Protection & Regulations	James Deye
Nonionizing Topics	Bhudatt Paliwal
Other Topics	Timothy Solberg
Book/Media Reviews	James Smathers
Editorials	John Horton
**Journal Production Team**	
Proofreader	Heather Shand
Copy Editor	Betty R. Robinson
Layout Editor	Laura Shand
Manuscript Manager	Patricia Walker

